# Supplementary material for: Metagenomic binning with assembly graph embeddings
Source: Bioinformatics. 2022 Aug 16;38(19):4481–7. doi: 10.1093/bioinformatics/btac557 (PMC9525014; doi:10.1093/bioinformatics/btac557)
Supplement: btac557_supplementary_data [file btac557_supplementary_data.zip › amber outputs.zip!/amber outputs/simHC/genome/completeness_minus_contamination.pdf]

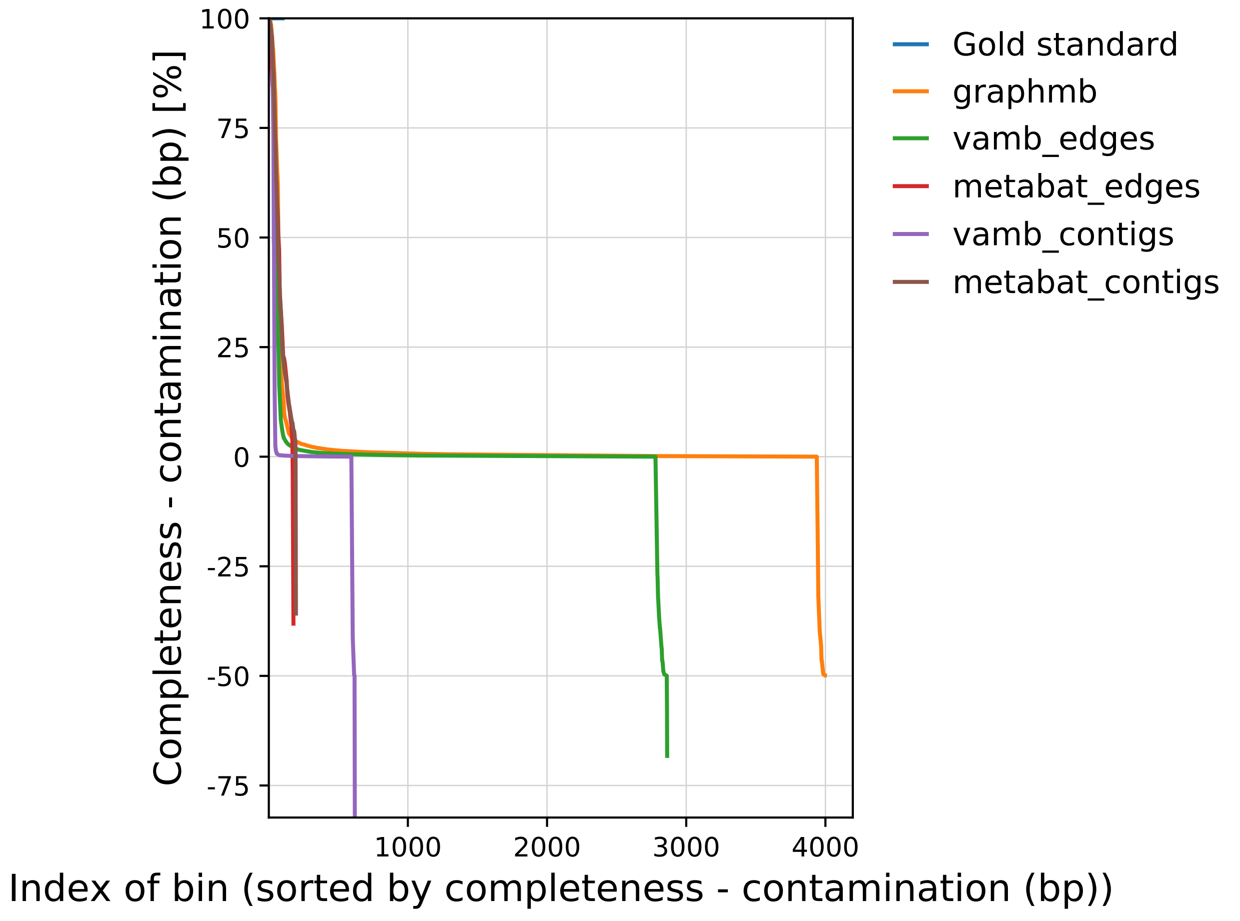
Gold standard
graphmb
vamb_edges
metabat_edges
vamb_contigs
metabat_contigs
Completeness - contamination (bp) [%]
Index of bin (sorted by completeness - contamination (bp))
100
75
50
25
0
-25
-50
-75
1000
2000
3000
4000
